# The Online Coalition Game: A tool for online interactive coalition formation research

**DOI:** 10.3758/s13428-021-01591-9

**Published:** 2022-01-24

**Authors:** Joeri Wissink, Ilja van Beest, Tila Pronk, Niels van de Ven

**Affiliations:** 1 Department of Social Psychology, Tilburg University, Warandelaan 2, 5037 Tilburg, AB Netherlands; 2 Department of Marketing, Tilburg University, Warandelaan 2, 5037 Tilburg, AB Netherlands

**Keywords:** coalition formation, negotiation, online interactive experiment, open access, oTree

## Abstract

In this paper, we present the Online Coalition Game (OCG): an open-source tool written for the open-access research platform oTree that enables high-powered interactive coalition formation experiments. Besides containing a tutorial on conducting and configuring studies using the OCG, we discuss two previous implementations. With these examples, we demonstrate that online use of the OCG provides the benefits of large sample sizes and fast data collection, while leading to convergent and robust findings. Moreover, we show that small changes in the experimental setup offer interesting opportunities to expand coalition formation theory by including insights from, amongst others, literature on bargaining, ostracism, and communication, and vice versa.

Coalition formation is a ubiquitous phenomenon. Coalitions can be seen at different levels of society: employees form (informal) coalitions to further their own goals in organizations (e.g., Stevenson et al., 1985), political parties form governments to rule countries (e.g., Bäck & Dumont, 2008), and companies form joint ventures to increase their market share or potential (such as the large KLM, China Southern, Xiamen and Air France joint venture; https://news.klm.com/successful-joint-venture-expanded/).

Despite this ubiquity, coalition formation research—a booming field from the 1950s until the 1980s—has not received much attention in contemporary social psychological theorizing. One reason for this decline may be the relative complexity of conducting these studies in which more than two participants interact in real time, combined with the increased awareness of needing large sample sizes to achieve sufficient statistical power. The latter may be exacerbated in coalition formation research, where the coalition that is formed is often the unit of analysis, and a group of at least three participants is needed for one observation. Despite the emergence of platforms that allow online real-time interactions between participants (e.g., Balietti, 2017; Chen et al., 2016; Giamattei et al., 2020; Hawkins, 2015; Molnar, 2019; Pettit et al., 2014), and despite the fact that computer-mediated coalition formation experiments have been available for almost half a century (Rapoport & Kahan, 1974)**,** up until now, there has been no open-source application that allows for structured online real-time coalition formation experiments.^1^

Instead of taking the intricacies of multiple negotiation partners and the formation of coalitions into account, the majority of negotiation studies rely on (dyadic) studies in which coalitions cannot be formed (Bendersky & McGinn, 2010). An example of why it is important to take the possibility of multiple negotiation partners into account can be found in a study investigating the effect of displaying anger on negotiation outcomes. Whereas displaying anger during dyadic negotiations seems to lead to higher payoffs, displaying anger during coalition bargaining often drives the target of the anger display away and into a coalition with a third negotiation partner (van Beest et al., 2008). Similarly, although deception may increase one’s outcomes in two-party negotiation, the use of deception is likely to lead to exclusion from coalitions (van Beest et al., 2011). This illustrates that ignoring the possibility of coalition formation in one’s research designs might mean that one’s findings are not generalizable to more complex settings.

To facilitate the investigation of these more complex multi-party negotiations, we have developed the Online Coalition Game (OCG): an application for conducting (online) interactive coalition formation research written for the open access platform oTree (Chen et al., 2016). Using the OCG allows researchers to conduct high-powered coalition formation experiments in which participants bargain online and in real-time about inclusion in a coalition and the division of the payoffs generated by a coalition.^2^ Moreover, making small changes in the experimental setup of the OCG offers interesting opportunities to expand coalition formation theory by including insights from, amongst others, literature on bargaining, ostracism, communication, and vice versa.

The rest of the paper is structured as follows. First, we will describe the two major coalition formation procedures we have implemented in the OCG. Next, we provide an overview of the choices researchers have in configuring different aspects of an OCG experiment and the insights these different configurations can provide. Subsequently, we provide some best practices when conducting an OCG experiment and describe a few measures we have taken to deal with the online and interactive nature of OCG experiments. After this, we discuss the results of two projects. One project demonstrates that the OCG leads to robust and converging findings by showing the robustness of a key finding in coalition formation—the Strength-is-Weakness effect (e.g., Vinacke & Arkoff, 1957; Wissink et al., 2021a)—and by comparing results obtained in a traditional laboratory at a university and results obtained using an Amazon Mechanical Turk (MTurk; https://www.mturk.com) sample. We also discuss one project (Wissink et al., 2021b) that demonstrates how a simple change in the OCG allowed us to investigate the effect of a moderator on established coalition formation findings and simultaneously extended accountability theory (Konow, 1996, 2000) to coalition formation settings. Finally, we discuss some future possible implementations of the OCG, for example to study threats to the need to belong due to exclusion (Baumeister & Leary, 1995; Williams, 2007) or phantom BATNAs (Best Alternative to a Negotiated Agreement) (Pinkley et al., 2019). Appendices A and B provide a short overview of oTree and explain how to use it to conduct a study using the OCG. Appendix C provides an overview of the different parameters used to configure the OCG. Finally, Appendix D provides the most important output variables.

## Experimental coalition formation protocols

Coalition formation has been defined as ‘the joint use of resources to determine the outcome of a decision in a mixed-motive situation involving more than two units’ (Gamson, 1964, p.85). It thus entails situations in which at least three individuals (or groups) strive to attain an outcome that they cannot attain individually, but in which individual gains cannot be maximized when all individuals cooperate. Hence, whereas in dyadic bargaining situations the focal questions are often *whether* and *how* bargainers reach a negotiated agreement, in coalition formation situations the focal question often is *who* reaches an agreement and who ends up being excluded from it.

In order to experimentally study these questions, coalition researchers have devised simple weighted majority games (see Komorita, 1984) such as the political convention (Gamson, 1961b) and landowner paradigms (van Beest et al., 2004a). Although they differ in context, these paradigms share the same structure: participants receive an amount of resources that is insufficient to obtain a monetary payoff by themselves, but which allows them to form coalitions in which their pooled resources are sufficient to obtain the payoff together. Importantly, they do need to form a consensus on how to allocate this payoff among the members of the coalition.

The simplest simple weighted majority game is one in which three participants each have one resource, and need to form a coalition with at least two resources (a threshold often referred to as to as a *decision point*) to be able to allocate a sum of money—a game referred to as a 2(1-1-1) simple weighted majority game. Often, however, bargainers differ in resources, such as in the common 5(4-3-2) game, in which three participants receive four, three, and two resources, respectively, and need to form a coalition with at least five resources. The way bargainers negotiate with each other, however, differs across different bargaining protocols. The OCG implements two dominant bargaining protocols for three-person coalition formation studies: the one-step Komorita and Meek (1978) display procedure and the more dynamic Kahan and Helwig (1971) procedure. This enables researchers to replicate classic (e.g., Komorita & Meek, 1978; Murnighan, 1978) as well as newer (e.g., van Beest et al., 2004b) coalition formation studies or adjust these protocols according to the needs of the study. Another reason for choosing these two protocols is that in both situations the participants make initial offers at the same time, meaning that initial offers that are independent of each other are collected for all participants. In these cases, bargaining results cannot be accounted for by differences in speed of decision-making.

### One-step protocol

**Fig. 1:**
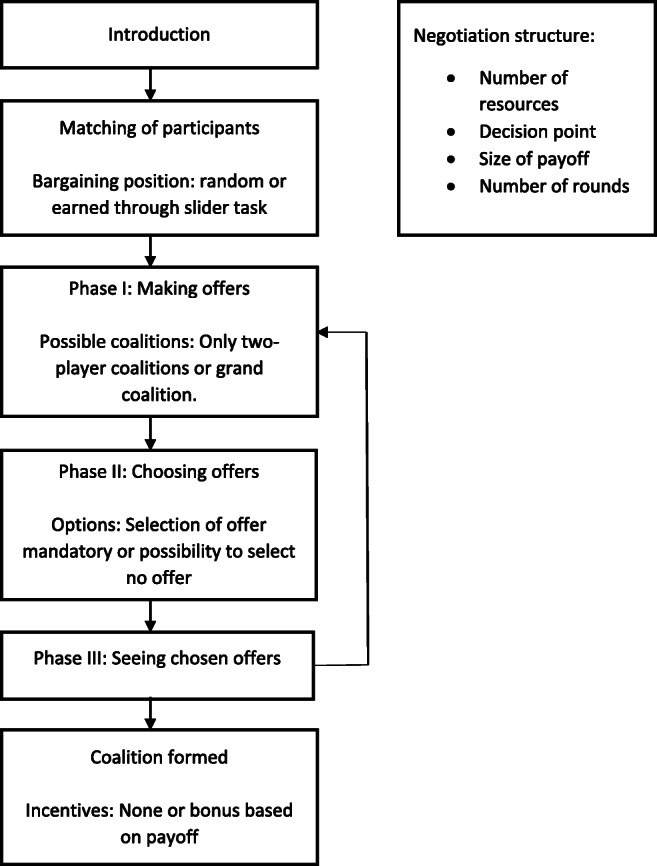
Phases and possible configurations of the one-step protocol

The Komorita and Meek (1978) display procedure is a one-step coalition bargaining protocol, meaning that when all members of a prospective coalition agree on how to allocate the payoffs the coalition is immediately formed. This bargaining protocol consists of three phases (see Fig. 1 for a visualization of the different phases and the choices researchers have in configuring the experiment).

In Phase I, all participants make a coalition offer. This offer consists of two things: a) with whom they want to form a coalition, and b) how they propose to allocate the payoff in this coalition. Coalitions can only be formed—and thus proposed—if a specified threshold (the decision point) is reached. For example, in the 5(4-3-2) simple weighted majority game, bargainers—for convenience labeled A, B, and C—hold four, three, and two resources, respectively, and the decision point is five resources, meaning that every coalition (AB with seven resources, AC with six resources, BC with five resources, and ABC with nine resources) can reach this threshold and can thus be formed.

**Fig. 2:**
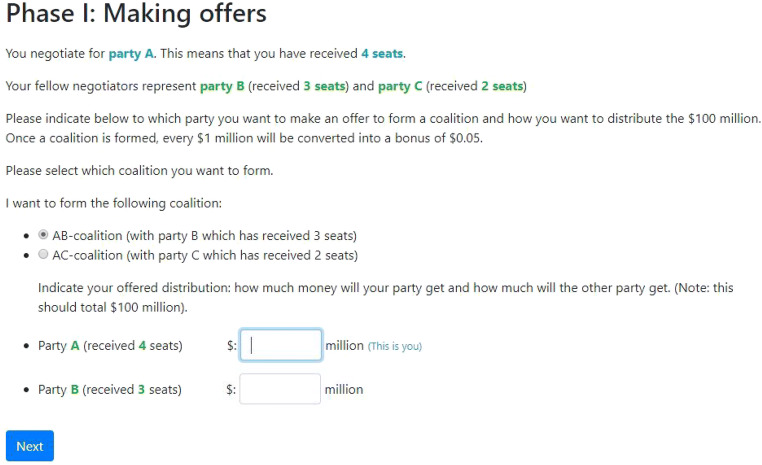
Screenshot showing Phase I of bargaining using the OCG

See Fig. 2 for the screen participants see when making an offer. In this example, we see the screen for bargainer A in a 5(4-3-2) simple weighted majority game. Note that in this example, the large ABC-coalition (the *grand coalition*) is prohibited, but the OCG has the option to allow its formation (see Appendix C on how to configure this).

In Phase II, participants see all offers that were made in Phase I. Participants then select one of the coalition offers that includes themselves. This could be either their own offer or an offer from another participant. Note that it is possible that participants have made the exact same offers in Phase I (e.g., B and C both propose a BC-coalition and the exact same equal split of the payoffs). In this case, this offer is displayed only once but it is indicated which participants have proposed this offer.

**Fig. 3:**
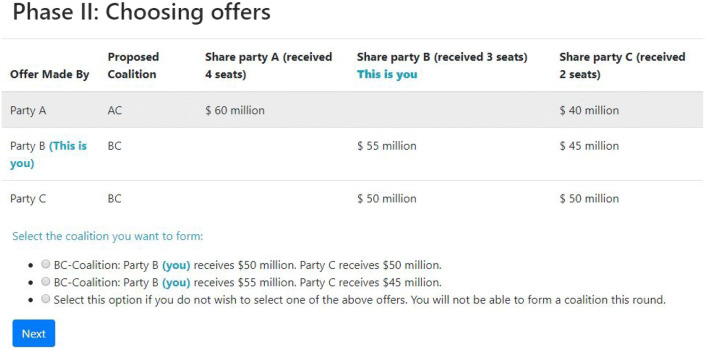
Screenshot showing Phase II of bargaining using the OCG

See Fig. 3 for the screen participants see when choosing an offer. In this example, we see the screen for bargainer B. In the previous phase, A has offered to form a coalition with C in which A proposed to allocate the payoffs so that A gets $60 million and C gets $40. Bargainers B and C both propose a BC-coalition, but B proposes to get $55 million and let C get $45 million, whereas C proposes an equal split of the payoffs. Note that the shown possibility to not select any coalition at all is an option that can be turned on or off (see Appendix C).

In Phase III, participants see who has selected which coalition offer. If all members of a proposed coalition have selected this offer, this coalition is formed and the payoffs are distributed as agreed upon by the members of the coalition. If no coalition is selected by all prospective members, a new round starts in which participants go through the same three phases. This process is repeated until a coalition is formed or when the last round, specified by the experimenter, is reached.

**Fig. 4:**
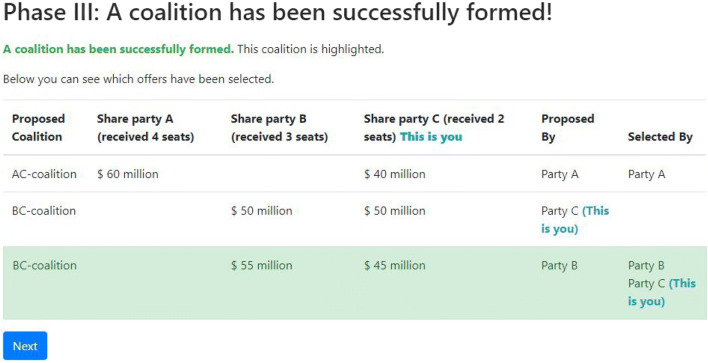
Screenshot showing Phase III of bargaining using the OCG

See Fig. 4 for the screen participants see when offers are chosen and a coalition has been formed. In this example, both B and C have selected B’s offer, meaning B gets $55 million and C gets $45 million. Only A has selected the self-made AC-offer and ends up excluded from the negotiated deal.

### Alternative offers protocol

**Fig. 5:**
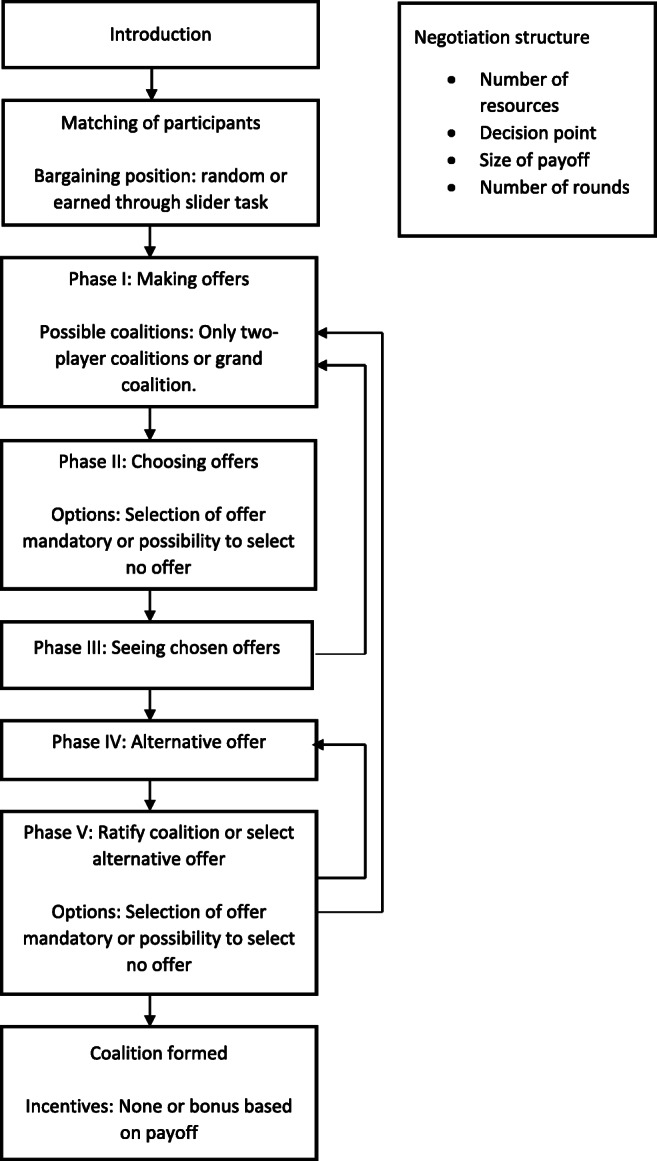
Phases and possible configurations of the alternative offers protocol

As a second bargaining protocol, we implemented the protocol from Kahan and Helwig (1971). Phase I and Phase II are identical to the ones used in the one-step procedure described above. After this, however, a few more steps are added, allowing those at risk at exclusion from a coalition to make an alternative offer, making the bargaining more dynamic than in the one-step procedure. For example, in the case presented in Fig. 4, bargainer A might realize (s)he is about to be excluded and might make a new, more attractive, offer to bargainer C in an attempt to prevent being excluded (see Fig. 5 for a visualization of the different phases and the choices researchers have in configuring the experiment).

In Phase III, participants see who selected which coalition offer. If no offer is selected by all prospective members, a new round starts in which participants go back to Phase I. If a coalition offer is accepted by all its prospective members, this does not directly lead to a coalition but to a *tentative* coalition. This means that the coalition is not binding, until the members of this tentative coalition confirm their preferences in step V.

In Phase IV, the coalition bargainer that is not in the tentative coalition has the opportunity to make an alternative offer to one of the bargainers in the tentative coalition. In this way, they may be able to tempt one of the bargainers to break away from the tentative coalition. If the earlier mentioned ABC-coalition is allowed, there is no excluded bargainer, meaning that no counteroffer is made. However, all bargainers will still need to ratify this coalition (or choose not to).

In Phase V, members of the tentative coalition have the option to ratify the tentative coalition and allocate the payoffs as agreed. However, the bargainer who has obtained an alternative offer may choose this alternative offer as well (or no offer at all, if this option is enabled). If this alternative offer is selected, the coalition proposed in the alternative offer becomes the new tentative coalition and another alternative offer can be made. This process will be repeated until a coalition is ratified or when the last round, specified by the experimenter (see Appendix C), is reached.

## Configuring an OCG experiment

In this section, we provide an overview of the choices researchers have in configuring different aspects of an OCG experiment, and the insights these different configurations can provide (see Figs. 1 and 5). For technical details on how to set these parameters and how to use oTree—the platform on which the OCG runs—see the appendices.

### Resources, decision point, and size of the payoff

Arguably, the most important decisions to make when configuring an OCG experiment are how many resources the different bargainers receive, how many resources are necessary to form a coalition (the decision point), and the size of the payoff available to a coalition.

Literature indicates that resources are used by bargainers to demand a share of the payoffs that is equitable to the proportion of resources they contribute to a coalition and that these equitable offers determine which coalitions are formed (e.g., Gamson, 1961a; Komorita & Chertkoff, 1973; Wissink et al., 2021a). Changing the relative (in)equality of resources thus allows researchers to test hypotheses regarding the role resources play in in shaping offers and the formation coalitions.

Different configurations of the resources and the decision point also allow for situations in which some bargainers have more alternatives than others have. For example, giving one bargainer three resources and two other bargainers both two resources and setting the decision point to four resources gives each bargainer two alternative coalitions they can form. However, setting the decision point to five resources limits the bargainers with two resources to only one possible coalition, whereas the bargainer with three resources has two alternatives, giving the latter more bargaining power. This thus also allows researchers to test hypotheses regarding the effect of bargaining power (e.g., Gamson, 1964; Komorita, 1974).

Finally, different configurations of resources and size of the payoff make it easier or more difficult for bargainers to apply the equity rule. For example, in a 4(322) game, an outcome of $10 can easily be divided equitable in all coalitions, but $13 cannot. One might also make it easier for some coalitions to form equitable allocations than for others: in a 5(432) game, $70 can be equitably divided between the bargainers with four and three resources, whereas $50 is more easily equitably allocated between the bargainers with three and two resources. Different configurations of resources and payoff allow for testing of hypotheses stating that the possibility of an equitable allocation is conducive to the formation of a coalition (van Beest et al., 2004b).

### Grand coalition

Besides manipulating which coalition can be formed by configuring resources and the decision point, researchers can choose whether only two-player coalitions can be formed or whether a *grand coalition* containing all three bargainers can be formed. Allowing the formation of grand coalitions could help extend research on the factors that promote the formation of oversized coalitions, such as consequences to excluded bargainers (van Beest et al., 2003) or Social Value Orientation (Van Beest et al., 2008)**.**

### Possibility to select no coalition

In Phase II, bargainers select the coalition offer they want to execute. Optionally, researchers can add the option for bargainers to select none of the coalition offers. Although this option is available in both bargaining protocols, it seems most useful in the alternative offers protocol, in which bargainers choosing between the tentative coalition and an alternative offer might want to have an exit option to go back to Phase I and make a new offer.

### Number of rounds

Researchers can determine the number of rounds bargainers have to form a coalition. In some situations, it could be desirable to set a high number in order to give ample room for bargaining but still have a way to end the experiment if bargainers fail to form a coalition repeatedly. In other experiments, the number of rounds might be varied between conditions as a manipulation of urgency or time pressure. This would, for example, allow researchers to study the assumption of bargaining theory (Komorita & Chertkoff, 1973) that bargainers will make more concessions when the pressure to form a coalition increases.

### Means of attaining resources

In classic coalition formation experiments, the number of resources bargainers hold is very often randomly obtained. Besides this option, the OCG also allows bargainers to earn a position with more resources by performing better on a real-effort task than the other bargainers. In a previous experiment (Wissink et al., 2021b), this allowed us to test whether accountability theory (Konow, 1996, 2000) is applicable to coalition formation. We describe this experiment in more detail below.

### Incentives

The OCG offers the possibility to give participants a bonus for the payoffs they have attained during the experiment. By setting a certain conversion rate of payoffs attained during the experiments to actual bonus payment, researchers are able to present a payoff that fits the scenario but hand out a small bonus amount that is proportional to the payoff obtained. When using the oTree MTurk integration, participation fees and bonus payments can be paid to all participants in one session with only one click. See https://otree.readthedocs.io/en/latest/ on how to set this up, but also see the possible downsides in Appendix B.

## Best practices in conducting an OCG experiment

In this section, we provide some best practices when conducting an OCG experiment and describe a few measures we have taken to deal with the online and interactive nature of OCG experiments.

### Introduction subsession

Due to the real-time nature of the OCG, we recommend starting a session with an introduction subsession in which participants are not yet matched (see Appendix A: Conceptual overview of oTree for an explanation on sessions and subsessions or the excellent oTree documentation: https://otree.readthedocs.io/en/latest/.). As there will be online participants that start a session but will not finish it, immediately matching participants will increase the chances that one participant in a group will make it impossible for the other (matched) participants to continue. By matching after the introduction subsession, only participants that have already read the first instructions will be matched, increasing the chance that only active participants are grouped. In the two preset session configurations, we have added the instructions in a first subsession prior to the bargaining subsession.

### Matching participants

Participants are matched when three of them are in a waiting lobby we refer to as a *matching page*. To make sure participants do not wait indefinitely, it is possible to add a time limit to the matching page which allows participants to go to the end of the study and receive the participation fee after the timer runs out.

### Number of participants per batch

Part of configuring a session is indicating how many slots to open for participants. When using an online platform such as MTurk, we recommend collecting data for a single experiment in multiple batches of around 30 participants. We have noticed that substantially larger batches increase the differences in starting times between participants, making matching into triads more difficult. With too small batches, participant dropout might lead to too few participants to match. Also make sure to open up about double the number of slots in oTree than number of HITs on MTurk. When participants start a study but do not finish it, they will take up a slot in oTree, but if a HIT is not returned in a specified time, a new HIT will be opened by MTurk. Enough open slots in oTree should be available to accommodate participants that accept one of these new HITs.

### Timers

After participants are matched, idleness of one participant could potentially stall the advancement of other participants in the same group. To counteract this, we added timers to all pages between the matching and the formation of a coalition. When participants have not completed a page within the allotted time, they will be kicked from the program. Participants within the same triad of this kicked participant will be forwarded to the end of the experiment where they can obtain their participation fee. This unfortunately does lead to having to pay some participants, without their data being useful to a researcher. However, given that the fault is not with these respondents (but with another respondent dropping out) it is ethical to pay the participants who were doing everything right.

## Previous implementations

### Replication of the Strength-is-Weakness effect and converging evidence

The first implementations of the OCG were two replications of the Strength-is-Weakness effect in coalition formation, one in a laboratory and one on MTurk (studies and results described in detail in Wissink et al., 2021a, data-package available here: 10.34894/JXRELG). The Strength-is-Weakness effect is the observation that coalition bargainers with many resources are disproportionally often excluded from coalitions (e.g., Vinacke & Arkoff, 1957). In other words, bargainers with many resources are often excluded from a coalition, as the players in the two low-resource positions tend to form a coalition. This project served as the first high-powered replication of the effect. Due to the unavailability of access to a large online population, previous studies either had a small sample size (leading to questionable statistical power), or had to use within-subjects designs, which might be problematic due to suggested learning effects (e.g., Kelley & Arrowood, 1960).

In this project, we used the one-step procedure from Komorita and Meek (1978) in a 5(4-3-2) game in a landowner setting (van Beest et al., 2004b) in which player A had 4 acres of land, B had 3 acres and C had 2 acres. Two participants needed to form a coalition with at least 5 acres of land in which they agreed on how to divide the $100,000 the landowner paid them (of which every $1000 was converted to a real bonus of $0.05). We conducted a study with the OCG in both a laboratory setting at a university and one via MTurk.

As three possible coalitions could be formed, a Strength-is-Weakness effect would mean that the formation of coalitions would deviate from a distribution in which all three coalition would be formed in one-third of the cases—with a disproportionate number of BC-coalitions, excluding the bargainer with the most resources. Our laboratory sample of 156 psychology undergraduates (52 triads) had 80% power to find a medium to large effect size of *w* = 0.43 and we found an effect size of *w* = 0.78. The BC-coalition was formed in 67% of the cases, the AC-coalition in 29% and the AB-coalition in only 4%. In the MTurk sample of 240 US Americans (80 triads), we had 80% power to find a medium to large effect size of *w* = 0.35 and obtained an effect size of *w* = 0.72. The BC-coalition was formed in 65% of the cases, the AC-coalition in 27.5% and the AB-coalition in only 7.5%.

The comparison between the laboratory sample and the MTurk sample also allowed us to investigate whether the OCG leads to converging findings between different platforms. As both studies yielded highly similar results that were in line with previous literature—and comprehension across both studies was similar—in the laboratory 84% answered all comprehension checks correctly versus 83% on MTurk: these first uses of the OCG suggest that it leads to robust results that convergence in offline and online settings.^3^ Moreover, the online data collection clearly showed to be a more time-efficient method: in the laboratory we collected 52 triads in ten weekdays, whereas we collected 80 triads online in just a few hours spread across three workdays.

Besides the benefits, conducting online interactive research brings its own challenges (see Arechar et al., 2017). The main challenge we encountered in this project was matching participants and handling dropout. We paid a participation fee to participants that waited but did not get matched and to participants that dropped out due to an idle participant they were matched to. These participants did not provide data but did constitute 35% of our sampled participants. Note that the above percentage is likely dependent on various factors, such as the minimum amount of time participants need to wait on the matching page (in this study 5 min), the maximum amount of time participants have on one page when matched (in this study 2 min, see Appendix C for an explanation on how to set timers), and familiarity with the interface. As an example of the latter, in this study, 12 participants—and thus also their 24 matched participants—got stuck due to trying to make offers in an incorrect format and thus dropped out due to the time limit. In the current version of the OCG, we have added a mandatory practice offer to avoid this issue.

### Adding moderators and extending accountability theory

Our second implementation demonstrates how a simple change in the configuration of the OCG allowed us to test a moderator of the Strength-is-Weakness effect whilst at the same time broadening the scope of accountability theory (Konow, 1996, 2000). In this project, we manipulated how bargainers attained their resources: through random assignment or through a real-effort slider task (study and results described in detail in Wissink et al., 2021b, data-package available here: 10.34894/FCLGKP).

According to accountability theory, one’s fair allocation should ‘vary in proportion to the relevant variables that he can influence (e.g., work effort but not according to those that he cannot reasonably influence (e.g., a physical handicap)’ (Konow, 2000, pp. 1973–1974). Ample research shows that people are in favor of equitable allocations based on earned input but not based on randomly received input (e.g., Frohlich et al., 2004; Lee & Shahriar, 2016; Oxoby & Spraggon, 2008; Ruffle, 1998). Our hypothesis was that this phenomenon extends to inclusion in coalitions: when bargainers have attained their resources by exerting effort, bargainers with more resources—who generally ask for an equitable share based on their resources—are approached and included more often than bargainers with more resources that have randomly received their resources, despite the possibility of avoiding these bargainers and opting for a cheaper coalition.

To test this, we again used an incentivized 5(4-3-2) game and one-step protocol embedded in a scenario in which participants bargain for municipal parties that have four, three, and two seats, respectively, and in which five seats were necessary to distribute allocate the budget of $90 million (with each $1 million being converted to a $0.05 bonus). In a *Random Resources* condition (170 triads), participants were randomly assigned their resources. In the *Earned Resources* condition (171 triads), participants who performed better on a real-effort slider task obtained more resources than those that performed worse (see Appendix C for instructions on how to implement the slider task). More first offers were made to those with more resources when resources were earned (60.8%) than when they were randomly received (51.8%), *OR* = 1.45. Whereas bargainers with the most resources were included more often when resources were earned (67.3%) than when they were assigned randomly (57.6%), *OR* = 1.51, this difference was not statistically significant. Answers on a post-bargaining questionnaire complemented this data from first offers and formed coalitions, thereby suggesting a possible explanation for these outcomes. Perceptions that bargainers with more resources deserve to be included (measured on a questions with a seven-point response scale) were higher in the Earned Resources condition (*M* = 4.48, *SD* = 1.77) than in the Random Resources condition (*M* = 3.60, *SD* = 1.89), *d* = 0.48. However, these perceptions were always higher for the bargainers with most resources themselves (*M* = 4.50, *SD* = 1.87) than for those with 3 resources (*M* = 3.86, *SD* = 1.82), and 2 resources (*M* = 3.77, *SD* = 1.88), *η*2 = .01. This suggests that bargainers with most resources did receive more first offers in the Earned Resources condition because they were considered deserving of inclusion, but that their own (even more inflated) sense of deservingness led them to bargain in a self-interested way that did not promote actual inclusion. It also shows how a survey with questions on people’s thoughts and perceptions might complement the actual bargaining behavior to provide further insights into the processes behind the negotiation choices respondents make.

## Future avenues

The second project described above demonstrates that the OCG enables the extension of theories to coalition formation settings, which allows a focus on the dynamics of inclusion and exclusion. Besides the possibility of incorporating real-effort slider tasks, the availability of a computerized coalition formation task opens up interesting avenues for research. For example, using the oTree chat functions can be used to further study the role of communication channels in coalition bargaining (e.g., Swaab et al., 2009).

Moreover, varying whether an inclusive coalition including all participants can be formed (see Appendix C on how to configure this) could inform about dynamics regarding inclusion and exclusion. As such, the OCG could be used to investigate whether exclusion from the bargaining table leads to similar threats to the need to belong as social exclusion typically does (Baumeister & Leary, 1995). Perhaps such exclusion hurts even if it is financially beneficial (similar to €yberball studies, van Beest & Williams, 2006), and people make suboptimal coalition offers in order to avoid exclusion. Moreover, whereas we know a lot about the consequences of exclusion (see Williams, 2007), the OCG could complement this research by studying processes leading to inclusion and exclusion.

The OCG could also be used to further investigate the effect of phantom BATNAs (Best Alternative to a Negotiated Agreement) (Pinkley et al., 2019; Pratkanis & Farquhar, 1992). In dyadic studies, BATNAs are often a static, predetermined payoff that participants get if they are not able to reach a negotiated outcome. Phantom BATNAs, on the other hand, are uncertain alternatives that may or may not materialize. Within the coalition formation framework, the bargainer to whom a participant does not yet send an offer to could be seen as a phantom BATNA: someone to make an offer to if one’s preferred coalition turns out to be less profitable than expected. As such, phantom BATNAs could be conceptualized in terms of bargaining alternatives by manipulating the number of resources bargainers hold and the number of resources a coalition needs to have to access the payoffs. Whereas research on phantom BATNAs shows that having more opportunities leads to higher power perceptions and higher bargaining performance (Pinkley et al., 2019), these hypotheses could be tested in the realm of coalition formation in which driving a hard bargain may backfire as it can lead to exclusion.

Finally, the OCG framework could be extended to, for example, conduct experiments with more than three bargainers, or to conduct multivalued studies (i.e., quota games) in which different coalitions yield different payoffs. Although this is not possible within the current version of the OCG, programming enthusiasts with knowledge of Python, HTML and Django, should be able to build these features on top of the existing code.

## Conclusions

In this paper, we presented the Online Coalition Game: an open-source tool enabling high-powered interactive coalition formation research. We demonstrate that online use of the OCG provides the benefits of large sample size and fast data collection, while leading to robust findings that converge between offline and online settings. Moreover, we show that the parameters of the bargaining setting can be tweaked, which allows interesting opportunities to expand coalition formation theory by including insights from, amongst others, literature on bargaining, ostracism, and communication.

## Appendix A: Conceptual overview of oTree

The OCG is programmed to run on oTree (Chen et al., 2016), an open-source platform that enables researchers to conduct real-time interactive experiments in laboratories and over the internet. The reason for this is that oTree’s architecture makes it highly suitable for interactive bargaining. Although oTree is not the only open-source platform capable of online interactive experiments (see for example Balietti, 2017; Giamattei et al., 2020; Hawkins, 2015; Molnar, 2019; Pettit et al., 2014), oTree is currently the only one supporting coalition experiments through the OCG. Moreover, oTree is under constant development, both as a platform (see https://otree.readthedocs.io/en/latest/misc/version_history.html) and in terms of new applications which could be used in the same session as the OCG to gain a richer understanding of bargainers. The OCG can for example be linked to an oTree implementation of the Social Value Orientation Slider Measure (Crosetto et al., 2019) or applications for time preference elicitation (Rose & Rose, 2019).

Whereas an in-depth tutorial of oTree is beyond the scope of this tutorial (see https://otree.readthedocs.io/en/latest/ for extensive documentation), we provide a conceptual overview here.

**Sessions and subsessions.** When conducting an experiment, multiple participants are recruited in one *session*. In this session, participants may complete multiple tasks that have their own applications (apps). Every app included in the session will have their own *subsession*. For example, when conducting a study using the OCG, participants usually go through two subsessions: first they read the introduction (subsession 1) after which they bargain (subsession 2). More subsessions can be added if desired, for example one with a post-bargaining questionnaire or another bargaining protocol.

**Participants, players, and groups.** When participants start a session, they can be identified throughout the entire session by a *participant* code. The word *player* refers to a role a participant takes while bargaining. In the OCG, *player* refers to the three different bargaining positions bargainers can have: A, B, and C. When participants are matched to interact with each other, they are put together in a *group*. In the OCG, three participants will form a *group* of three *players*.

**Pages and waitpages.** A (sub)session consists of different *pages* and *waitpages*. These are the different screens participants go through when completing a session. *Pages* refer to screens in which participants have to do something, be it reading a text, making an offer, or answering some questions. *Waitpages* are pages on which participants wait until all members of one group arrive on this page. These *waitpages* are used when input from all participants is needed for a next step in the application. In the OCG, there are *waitpages* in between the different phases in the bargaining protocol. For example, participants cannot go to the selection of offers (Phase II) until all participants have made their offer. *Waitpages* can also be used to match *players* in a *group*. The first *page* of the two OCG bargaining apps are such a *waitpage*.

**Hierarchy of objects.** The above objects (*session, subsession, group, players,* and *pages*) are all nested. In a *session* there can be multiple *subsessions*, which contain *groups*, which contain *players*, who go through multiple *pages* and *waitpages*. This also means that it is relatively easy to conduct a certain computation for all *players* in a *group* or for all *groups* in a session. See the conceptual overview section in the oTree documentation for more technical details.

## Appendix B: Installation and set-up of the Online Coalition Game

**Installing the Online Coalition Game.** After installing oTree (see oTree documentation), and starting a new project, download the Online Coalition game here: https://github.com/JoeriWissink/OnlineCoalitionGame. Copy the folders *Online_Coalition_Game, Online_Coalition_Game_Alternative_Offer, Online_Coalition_Game_Introduction,* and *Online_Coalition_Game_ _Alternative_Offer_Introduction* to your project folder and copy and paste the *SESSION_CONFIGS* from the *settings.py* file into your own *settings.py* file.

**Setting up a server.** Although studies can be tested on local devices, use of a web server is necessary for conducting an online study. The easiest way to do this is hosting the study on Heroku—a cloud hosting provider (https://www.heroku.com/). Another possibility is setting up a dedicated server. See the oTree documentation for more information.

**Configuring a session.** Experimenters interact with the server using an *admin* interface. Launching a new session can be done at the *Sessions* page. Here one of the sessions configured in the *settings.py* file located in your oTree project folder can be launched. In this file, we have already preset two session configurations under *SESSION_CONFIGS*, one using the one-step protocol and one using the dynamic protocol. Both include an *Instructions* subsession before the *Bargaining* subsession. It is also possible to add a session configuration under *SESSION_CONFIGS*. Moreover, the session can be configured in the admin *Sessions* page after selecting one of the session configurations. See Appendix C: Configuring the Online Coalition Game for a description of the different parameters of the OCG.

**Reaching participants.** After having created a session, access to the experiment can be given by distributing the session URLs found under the *Links* tab. When conducting a study on MTurk, the *Session-wide link* can be shared in the HIT. When conducting a laboratory session, it might be easier to use stable URLs for each computer. In this case, a session can be launched, and configured, on the *Rooms* page in the admin interface instead of the *Sessions* page and the links to these stable URLs can be placed on the computer desktops. As oTree is totally URL based, all participants need is an internet capable electronic device.

Alternatively, researchers can make use of the oTree MTurk integration (see oTree documentation: https://otree.readthedocs.io/en/latest/). This makes it possible to launch HITs from the oTree admin environment and easily pay earned bonuses to participants. A downside of this approach is that this precludes integration with CloudResearch (https://www.cloudresearch.com; formerly known as TurkPrime) and thus its possibility to filter out suspicious IP addresses and locations.

NB: Whereas the experiment functions well across platforms and browsers, we encountered an exception in which a real-effort slider task did not function properly on Internet Explorer and Microsoft Edge. Using JavaScript, we managed to detect participants using these browsers and prompted them to use another browser (see Appendix C).

**Monitoring participants.** After having launched a session, participants’ progress can be monitored under the *Monitor* tab. This tab shows how many participants are currently in the session and where they are in the session.

**Paying participant.** When participants are finished, they are forwarded to a screen displaying a randomly generated completion code, which they can enter when submitting their HIT on MTurk. This code also allows linking their HIT to the bonus they are entitled to receive. When using the oTree MTurk integration, oTree automatically records which participants have finished the experiment as well as the bonus they earned. However, see the downside of using this integration above.

**Downloading data.** After data is collected, it can be downloaded in the *Data* tab of a session or the general *Data* page displaying all sessions. See Appendix D for an overview of the most important outcome variables.

## Appendix C: Configuring the Online Coalition Game

When starting a session of the online coalition game, there are a number of parameters that can be configured.

**Protocol.** Which bargaining protocol is used is determined by the chosen session configuration. To use the one-step protocol use *Online Coalition Game One-Step Protocol* and for the more dynamic alternative offer protocol use *Online Coalition Game Dynamic Protocol.*

**Player resources and decision point.** Configuring *resources_player_A, resources_player_B,* and *resources_player_C* determines the number of resources the different bargainers in a triad hold. With *decision_point*, a threshold can be set, determining which coalitions can form a coalition. NB: While setting the resources and decision point, take into account that the OCG does not support situations in which a participant cannot be part of any coalition.

**Grand coalition.** Setting *grand_coalition* to True will enable the formation of a three-player coalition including all three participants. Setting it to False will only permit two-player coalitions.

**Total payoff.** Configuring *total_payoff* will set the size of the payoffs participants bargain for. Note that only integers are accepted.

**Payoff conversion.** Configuring *payoff_conversion* sets the conversion rate from obtained payoff to bonus payment.

**Incentives.** When *incentives* is set to True, participants will receive information about the conversion rate and earned bonus. If set to False, this information is left out. NB: Make sure to set *USE_POINTS* to False in *settings.py* to correctly display the bonus in terms of money.

**Base fee.** Configuring *base_fee* sets the amount of money participants receive for reaching the end of the game, either after forming a coalition, having waited long enough at the matching page, or dropping out due to an interaction partner being kicked.

**Select none.** When *select_none* is set to True, participants will have the option to choose no coalition offer at all in Phase II: Selecting offers. When it is set to False, this option is not available.

NB: When using the dynamic bargaining protocol and grand coalitions are allowed, setting *select_none* should be set to True to give bargainers an option to retreat from a tentative ABC-coalition.

**Timeout time.** Configuring *timeout_time* sets the number of seconds participants are allowed to spend on each page between the matching and the formation of a coalition.

**Earned.** When *earned* is set to True, participants will obtain their bargaining position based on their relative performance on three rounds of a real-effort slider task. Within a triad, the participant that completed most sliders obtains position A, the participant that came in second position B, and the one that came in last position C. When *earned* is set to False, participants will randomly obtain one of the three positions. When used, a test round is added to the instructions.

NB: The sliders do not work well when using Internet Explorer or Microsoft Edge. To prevent participants with these browsers from taking the study, a script has been added to the first page of the introduction apps prompting participants to use a different browser.

**Slider time.** Configuring *slider_time* sets the number of seconds participants will have to complete as many sliders in each of the three rounds.

**Comprehension check.** When *comprehension_check* is set to True, participants will receive three comprehension questions concerning the setting.

**Leave matching.** When *leave_matching* is set to True, participants will be forwarded from the matching page to the end of the study after the time limit set in *leave_timer*. NB: Using this function requires a landing app in which the following code is added to the last page in views.py (as done in the two introduction apps of the Online Coalition Game):

*def before_next_page(self):*

*import time*

*self.participant.vars['wait_page_arrival'] = time.time()*

**Leave timer.** Configuring *leave_timer* sets the number of seconds participants wait to be grouped before being forwarded to end of the study.

**Number of rounds.** In the OCG, the number of rounds—configured with *num_rounds* in *Online_Coalition_Game\models.py* or *Online_Coalition_Game_Alternative_offer\models.py,* not via the session configuration—determines the maximum number of rounds participants have to form a coalition. When a coalition is formed, the remaining number of rounds will be skipped. When a coalition is not formed within the number of rounds set, participants will be forwarded to the end of the experiment.

## Appendix D: Output variables

After an experiment, oTree provides a large dataset with one row per participant and many variables for each round. In Table 1, we outline the key variables necessary to understand the bargaining process and outcomes.

**Table 1:** Output variables of the Online Coalition Game

**Level**	Variable name	Stores
**Player**	position	Player position (A, B, or C)
	proposed_coalition	Name of the coalition proposed in Phase I
	allocate_to_player_A/B/C	Proposed allocation to players A, B and C
	selected_coalition_name	Name of the selected coalition in Phase II
	tentative_selected_coaltion_name.	Name of the selected coalition in Phase II (dynamic protocol)
	selected_coalition_allocation_A/B/C	Allocations of the selected offer
	tentative_selected_coalition_allocation_A/B/C	Allocations of the selected offer (dynamic protocol)
	counter_proposed_allocation	Alternative offer (dynamic protocol)
	counter_allocate_to_player_A/B/C	Allocations of the alternative offer
	ratify_coalition	Participants’ choice to ratify the tentative coalition or select the alternative offer
	money	Obtained share of the budget
	completion_code	Participant’s completion code
**Group**	formed_coalition_name	Name of the formed final coalition
	payoff_A/B/C	Final payoffs of participants
	proposed_coalition_player_A/B/C	Proposed coalitions of players
	allocation_A_to_B	Proposed allocation of player A to player B
	selected_coalition_name_player_A/B/C	Name of the selected coalition in Phase II
	tentative_selected_coalition_name_player_A	Name of the selected tentative coalition in Phase II (dynamic protocol)
	selected_coalition_allocation_A_player_B	Allocation to player A in the coalition selected by player B
	tentative_selected coalition_allocation_ A_player_B	Allocation to player A in the coalition selected by player B (dynamic protocol)
	tentative_formed_coalition_name	Name of formed tentative coalition (dynamic protocol)
	counter_proposed_coalition_name	Name of the proposed alternative coalition (dynamic protocol)
	counter_proposed_allocation_to_player_A/B/C	Proposed allocation in the alternative offer (dynamic protocol)
	ratify_coalition_A/B/C	Choice of players to ratify tentative coalition or choose the alternative offer (dynamic protocol)
	new_tentative_formed_coalition_name	Name of the new tentative formed coalition (dynamic protocol)
	new_tentative_payoff_A/B/C	Allocations in the new tentative payoff (dynamic protocol)
	round_begin	Phase at which the round begins (dynamic protocol)
	round_end	Phase at which the round ends (dynamic protocol)
**Identifiers**	participant.code	Unique participant ID in a session
	group.code	Unique group ID in a session
	session.code	Unique session ID

## Footnotes

1 Alternatives to the Online Coalition Game are paid services (e.g., https://idecisiongames.com/promo-home) or bargaining through online communication platforms (e.g., Zoom). The former, however, limits researchers with a smaller budget and does not allow researchers to modify the experiments to their needs. Whereas communication platforms allow for a rich (visual) interaction, the Online Coalition Game provides a more structured and controlled environment in which bargaining decisions, such as chosen negotiation partner and offer, are automatically recorded.

2 Note that the Online Coalition Game has been developed to conduct simple weighted majority games in which bargainers are assigned resources and need to combine these in a coalition to attain a fixed sum payoff (see Komorita, 1984). However, by not displaying these resources to participants it is possible to conduct experiments that do not focus on the resources bargainers hold. Moreover, with a bit of additional programming, it is possible to conduct multivalued experiments, such as quota games, in which different coalitions lead to different payoffs (e.g., Funk et al., 1980) or experiments involving resources and quotas (Komorita et al., 1989).

3 Note that this convergence between laboratory setting and online setting does not speak to its external validity. As previous scholars have argued, factors such as size of the stakes, experience, WEIRDness of the sample and anonymity may lead behavior outside of experiments to differ from behavior obtained in experiments (Henrich et al., 2010; Levitt & List, 2007a, 2007b). Studies on governmental coalition formation in which a Strength-is-Weakness effect is observed (Bäck & Dumont, 2008; Warwick, 1996), do suggest convergence between coalitional behavior and outcomes inside, and outside, laboratories.
